# The mayfly *Neocloeon triangulifer* senses decreasing oxygen availability (*P*_O_2__) and responds by reducing ion uptake and altering gene expression

**DOI:** 10.1242/jeb.247916

**Published:** 2024-11-28

**Authors:** Jamie K. Cochran, David B. Buchwalter

**Affiliations:** Department of Biological Sciences, North Carolina State University, Raleigh, NC 27695, USA

**Keywords:** Osmoregulation, Oxygen sensing, Hypoxia, *P*_crit_, Gene expression

## Abstract

Oxygen availability is central to the energetic budget of aquatic animals and may vary naturally and/or in response to anthropogenic activities. Yet, we know little about how oxygen availability is linked to fundamental processes such as ion transport in aquatic insects. We hypothesized and observed that ion (^22^Na and ^35^SO_4_) uptake would be significantly decreased at O_2_ partial pressures below the mean critical level (*P*_crit_, 5.4 kPa) where metabolic rate (*Ṁ*_O_2__) is compromised and ATP production is limited. However, we were surprised to observe marked reductions in ion uptake at oxygen partial pressures well above *P*_crit_, where *Ṁ*_O_2__ was stable. For example, SO_4_ uptake decreased by 51% at 11.7 kPa and 82% at *P*_crit_ (5.4 kPa) while Na uptake decreased by 19% at 11.7 kPa and 60% at *P*_crit_. Nymphs held for longer time periods at reduced *P*_O_2_ _exhibited stronger reductions in ion uptake rates. Fluids from whole-body homogenates exhibited a 29% decrease in osmolality in the most hypoxic condition. The differential expression of *atypical guanylate cyclase* (*gcy-88e*) in response to changing *P*_O_2_ _conditions provides evidence for its potential role as an oxygen sensor. Several ion transport genes (e.g. *chloride channel* and *sodium-potassium ATPase*) and hypoxia-associated genes (e.g. *ldh* and *egl-9*) were also impacted by decreased oxygen availability. Together, the results of our work suggest that *N. triangulifer* can sense decreased oxygen availability and perhaps conserves energy accordingly, even when *Ṁ*_O_2__ is not impacted.

## Teaser

**Highlighted Article:** Responses of a mayfly species to reduced oxygen levels suggest they sense decreased oxygen availability and perhaps conserve energy accordingly, even when the rate of oxygen consumption (*Ṁ*_O_2__) is not impacted.

## INTRODUCTION

Dissolved oxygen (DO) is an important abiotic factor that varies naturally and plays roles in the distribution of aquatic species (Blaszczak et al., 2019; Eriksen, 1968; Hoback and Stanley, 2001; Huryn and Wallace, 2000). Hypoxia can occur naturally, most frequently at night when photosynthesis cannot counteract respiration, in stagnant waters and in areas with abundant organic matter or impeded flow (Resh et al., 2008). Anthropogenic activities exacerbate this and have led to growing concerns about hypoxia, globally. Nutrient runoff, eutrophication and changing global temperatures can all lead to hypoxic events (Diaz, 2001; Friedrich et al., 2014; Jenny et al., 2016a,b; Meire et al., 2013; Nixon, 1995). Hypoxia has the potential to impact all but the most hypoxia-tolerant aquatic species. Aquatic insects play disproportionately significant roles in these ecosystems and are widely used in ecological monitoring programs to make inferences about ecological conditions (Funk et al., 2006; Hawkins, 2006; Hawkins et al., 2000; Resh and Jackson, 1993). Oxygen availability is central to the energetic budget of these aquatic animals, and a lack of available DO can cause a cascade of effects.

Many organisms maintain a stable rate of oxygen consumption (*Ṁ*_O_2__) until they reach a point where their *Ṁ*_O_2__ becomes dependent on the amount of available oxygen (Brodersen et al., 2008; Lencioni et al., 2008; Pörtner and Grieshaber, 1993). The environmental oxygen level (*P*_O_2__) where an organism can no longer oxyregulate is traditionally called the *P*_crit_. While *P*_crit_ has been questioned as an indicator of hypoxia tolerance (Wood, 2018), it clearly has importance to the organism. For example, in the mayfly *Neocloeon triangulifer*, *P*_crit_ has been associated with significant upregulation of the hypoxia-responsive genes *egl-9* (*egg laying deficient*, an oxygen-sensing gene and modulator of HIF-1a activity) and *ldh* (*lactate dehydrogenase*, a hypoxia indicator) (Cochran et al., 2021). This suggests that *P*_crit_ represents a shift from aerobic to anaerobic metabolism in this species. As sufficient oxygen is necessary to convert nutrients into ATP, decreasing *P*_O_2__ results in decreased ATP synthesis, leading to a cascade of effects including decreased energy production, greater reliance on anaerobic respiration, and reallocation of energy to increase ventilation (Boutilier and St-Pierre, 2000; Verberk et al., 2020), specifically at *P*_O_2__ below *P*_crit_. As a result, aquatic insects (like other animals) must prioritize or allocate their energy expenditure. It is currently unknown where osmoregulation falls in terms of their energy prioritization.

Osmoregulation in freshwater is considered energetically expensive but physiologically crucial. Freshwater environments are typically strongly hypotonic to the organism's hemolymph, and therefore preventing excess ion loss and water uptake is critical in maintaining homeostasis (Silver and Donini, 2021). When ATP turnover is decreased in environments with decreased oxygen, it is unknown exactly how osmoregulatory function is impacted in aquatic insects. Studies in freshwater fish have found reduced sodium and water fluxes after exposure to severe hypoxia, with some differences existing between hypoxia-tolerant and less tolerant taxa (Giacomin et al., 2020; Iftikar et al., 2010; Onukwufor and Wood, 2020, 2018; Robertson et al., 2015; Wood et al., 2019, 2009, 2007; Wood and Eom, 2021). However, we are unaware of any studies that address osmoregulatory changes in freshwater insects, specifically at environmental oxygen levels that are reduced, but not low enough to cause physiological hypoxia.

Animals have evolved mechanisms to sense O_2_ concentration, with studies in large vertebrates revealing sophisticated neuronal circulatory and respiratory systems (Ma and Ringstad, 2012). However, little is known about similar systems in small invertebrates, such as aquatic insects. Studies in the nematode *Caenorhabditis elegans*, the fly *Drosophila melanogaster* and the crustacean *Procambarus clarkii* have identified guanylate cyclase (GC) subunits (e.g. *gcy-88e*) and hypoxia-inducible transcription factors (HIFs) as O_2_-sensing molecules (De Lima et al., 2021; Ma and Ringstad, 2012). The oxygen-sensing prolyl hydroxylase *egl-9* (also referred to as *hph* and *phd*) hydroxylates HIF to tag it for destruction, thereby abolishing signaling activity under normal conditions. Under hypoxic conditions, *egl-9* cannot hydroxylate HIF, which results in the accumulation of HIF and *egl-9* (Bishop et al., 2004; Cochran et al., 2021; Kim et al., 2017; Ma and Ringstad, 2012; Semenza, 2001; Shao et al., 2009; Shen and Powell–Coffman, 2003). In the mayfly *N. triangulifer*, measurement of HIF1α mRNA expression suggests it is constitutively expressed, with *egl-9* controlling HIF1α activity (K. S. Kim and D.B.B., unpublished data). However, to our knowledge there have been no other studies on oxygen sensing in aquatic insects to date.

Here, we used the lab-reared mayfly *N*. *triangulifer*, a parthenogenic species emerging as a useful model for ecological (Sweeney and Vannote, 1984), toxicological (Conley et al., 2014; Johnson et al., 2015; Kunz et al., 2013; Soucek and Dickinson, 2015; Sweeney et al., 1993; Xie and Buchwalter, 2011) and physiological studies (Chou et al., 2018; Cochran et al., 2021; Cochran and Buchwalter, 2022; Kim et al., 2017; Orr et al., 2021). Specifically, we exposed nymphs to ramps of dissolved oxygen, using *P*_crit_ estimates for *N. triangulifer* at 22°C (Cochran et al., 2021) to inform *P*_O_2__ set points. We used a radiotracer approach to ask whether Na and SO_4_ uptake rates were impacted by decreased oxygen availability (ranging from normoxia to hypoxia). We also measured the osmolality of fluids from whole-body homogenates and assessed the mRNA expression levels of hypoxia-responsive genes (*egl-9* and *ldh*) and genes related to ion transport (Orr et al., 2021) across the same environmental oxygen levels. Finally, we capitalized on our newly annotated *N. triangulifer* genome (NCBI GCF_031216515.1) to develop a qPCR probe for the oxygen-sensing *gcy-88e* gene.

## MATERIALS AND METHODS

### Animal collection and rearing

*Neocloeon triangulifer* (WCC-2 clone) were originally obtained from White Clay Creek in Pennsylvania by collaborators at Stroud Water Research Center (SWRC; Avondale, PA, USA) (Sweeney and Vannote, 1984). Nymphs were reared at North Carolina State University in 4-quart (∼4 l) glass Pyrex dishes filled with Artificial Soft Water (ASW) and lined with WCC periphyton plates at room temperature with gentle aeration, and a 14 h:10 h light:dark photoperiod. ASW was made with recipes from the United States Environmental Protection Agency (D. Mount, EPA, Duluth, MN, USA, 2017, personal communication) using a base of distilled water (∼17.8 MΩ) and laboratory-grade salts (Thermo Fisher Scientific, Waltham, MA, USA). Major ion concentrations of ASW (mg l^−1^) were determined to be: 27.5 Na, 15.5 S, 6.0 Ca, 2.2 Mg, 2.2 K by ICP-MS at NCSU's EATS laboratory. Mature nymphs (∼25 day rearing) were then removed from rearing dishes and used in experiments.

### Controlled oxygen experiments

The overall design of this experiment was to ‘step down’ larvae from normoxia (21 kPa) to progressively lower *P*_O_2__ (11.7, 8.5, 5.4 and 1.6 kPa), and measure changes in ion transport rates and the expression of a suite of genes related to oxygen sensing and ion transport. These methods and procedures to compare the responses of nymphs that had been stepped down sequentially with the responses of ‘naive’ nymphs that had previously been exposed to only 21 kPa are described in detail below. The *P*_O_2_ _treatments were based in part on a previous study (Cochran et al., 2021) that characterized population mean, maximum and minimum *P*_crit_ estimates in this species.

**Fig. 1.**
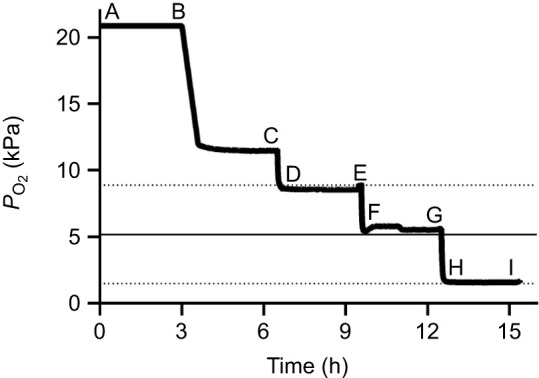
**Oxygen partial pressure (*P*_O_2__) and *Neocloeon triangulifer* nymph sampling points at 22°C.** Each letter denotes when nymphs were sampled for ion flux, osmolality and gene expression studies (as described in Materials and Methods, see Table S1). Letters D, F and H denote when a new cohort of naive nymphs was added to each exposure for a subset of the ion flux data. Dashed lines represent maximum and minimum critical oxygen partial pressure (*P*_crit_) estimates and the solid line represents mean *P*_crit_ from Cochran et al. (2021).

To achieve the desired *P*_O_2__ treatments, tanks of compressed nitrogen (Arc3^®^ gases UN1066; Fisherbrand™ Single-Stage Pressure Regulator with SCFH air flow meter) and compressed breathing quality air (Arc3^®^ gases UN1002; Harris^®^ model 425-125 Premium Single-Stage Pressure Regulator with SCFH air flow meter) were attached to a gas mixer, which distributed mixed air into a distribution manifold. Tubing from the manifold attached to 20 ml glass Wheaton^®^ liquid scintillation vials through lids with drilled bore holes to accommodate 1000 µl Fisherbrand™ Redi-Tip™ pipette tips and a smaller vent hole were used to control gas exchange with the room. Four of the 11 total chambers were used to monitor the experimental oxygen tension throughout the 15 h experiment (Fig. 1), with calibrated oxygen sensor spots and AutoResp™ software. Two of these monitoring chambers contained only water and two contained nymphs to ensure that their presence did not significantly alter the intended oxygen tensions in unmonitored experimental vials. Including the two monitored chambers, there were 9 total ‘experimental’ chambers that contained nymphs (see Figs S1 and S2 and Table S1 for a full overview). The vials contained 10 ml of ASW and were kept on the benchtop at room temperature. Temperature was constantly monitored using the AutoResp™ software in a separate vial kept on the benchtop. See Fig. S1 for setup.

Between 10 and 19 individuals were introduced into each of the 9 experimental vials (Fig. 1A) at the beginning of the experiment and exposed to 21 kPa for 3 h. A subset of animals (2–4 per vial) were collected after the 3 h exposure to 21 kPa (Fig. 1B) as controls. Eight individuals (from waters containing radioisotope) were collected for ion flux experiments (see ‘Ion flux’ section below for specific procedure), 5 were collected for osmolality measurements (see ‘Osmolality’ section below for specific procedure) and 10 were collected for gene expression (see ‘Gene expression’ section below for specific procedure). The remaining individuals were then ‘stepped down’ to 11.7 kPa and a similar subset of animals was collected from each vial at the end of the 3 h exposure period (Fig. 1C).

**Fig. 2.**
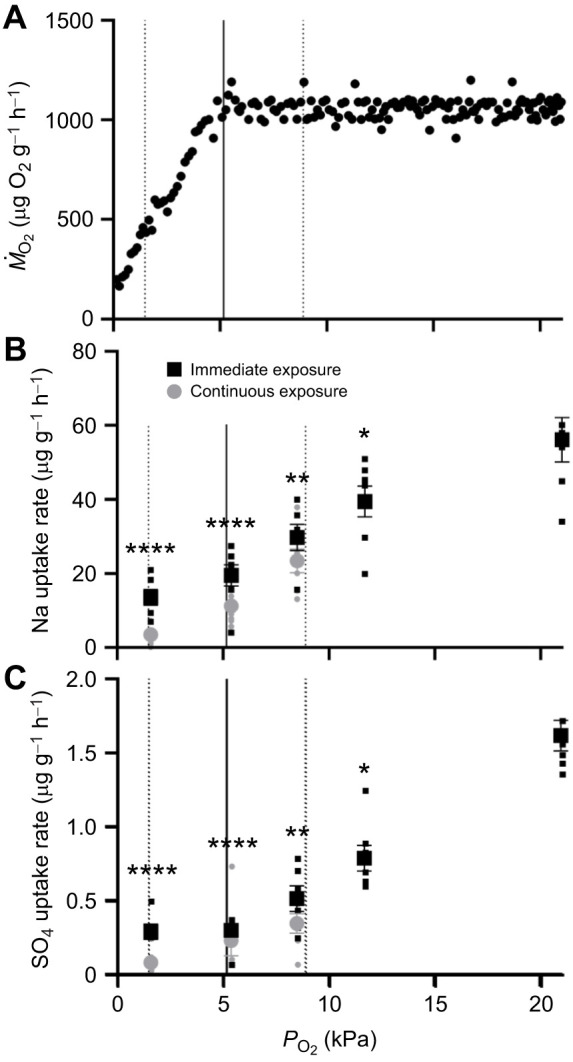
***Neocloeon triangulifer* ion flux rates at different *P*_O_2__.** (A) Representative metabolic rate (*Ṁ*_O2_) against *P*_O_2__ for one *N. triangulifer* individual from Cochran et al. (2021). Dashed lines represent maximum and minimum *P*_crit_ estimates, and the solid line represents mean *P*_crit_ estimated from 31 individuals at 22°C from Cochran et al. (2021). (B) Na and (C) SO_4_ uptake rates of *N. triangulifer* in depleted oxygen partial pressures (21–1.6 kPa) at 22°C (mean±s.e.m., *n*=7–8). Black squares represent nymphs that were introduced into chambers after the *P*_O_2__ was achieved, while gray circles represent nymphs that were introduced at the beginning of the experiment and experienced all prior *P*_O_2__ levels. Differences among treatments were assessed using a one-way ANOVA and Tukey's multiple comparisons test. All treatments were compared with the control (21 kPa) group. Asterisks represent significant differences compared with 21 kPa (*P≤0.05; **P≤0.01; ****P≤0.0001).

*P*_O_2__ was then decreased again to 8.5 kPa. To compare the responses of larvae that had experienced the previous *P*_O_2_ _drops with those of larvae that had only previously experienced normoxic waters, 10 normoxic individuals were added to one vial containing 10 ml of non-radioactive ASW and 8 individuals were added to the last vial containing 10 ml of radioactive ASW. This group of individuals was exposed instantaneously to a given *P*_O_2__ and did not experience any prior decreased oxygen regimes (Fig. 1D). These individuals were only used for a subset of the ion flux data (Fig. 2).

After a 3 h exposure to 8.5 kPa, a similar subset of individuals was collected from the vials of individuals exposed to all prior *P*_O_2__ (2–4 per vial). From the newly introduced vials of naive individuals, all 8 of the nymphs were removed (Fig. 1E).

*P*_O_2__ was then decreased again, this time to 5.4 kPa, and the same methodology was used. Eight naive individuals were introduced into the radioactive empty vial (Fig. 1F). After a 3 h exposure to 5.4 kPa, the same number of individuals was collected from all vials (Fig. 1G).

*P*_O_2__ was then decreased again, to 1.6 kPa, and the same methodology was used. Eight naive individuals were introduced into the radioactive empty vial (Fig. 1H). After a 3 h exposure to 1.6 kPa, all individuals were collected from all vials (Fig. 1I).

#### Ion flux

Dual-labeled radioactive experimental waters were made by spiking ^22^NaCl and Na_2_^35^SO_4_ (PerkinElmer, Billerica, MA, USA) into ASW to achieve exposure activities of 150–215 Bq ml^−1^. Exposures were measured with a Beckman LS6500 Multipurpose Scintillation Counter (Beckman Coulter, Brea, CA, USA) immediately before the experiments began.

Eight nymphs were removed from the radioactive exposure waters by gently pipetting them into a mesh strainer (collecting any residual radioactive water in a waste container) and gently blotting them dry. The nymphs were then rinsed in two consecutive water baths of ASW to remove loosely adsorbed ions from the exoskeleton. After rinsing, nymphs were blotted dry, weighed, and digested in 500 μl of Soluene 350 (PerkinElmer) in a 20 ml glass vial at 28°C. After 48 h, they were neutralized with 500 μl of glacial acetic acid and 12 ml of scintillation cocktail (PerkinElmer Ultima Gold uLLT).

Uptake rates were calculated as the slopes of linear regressions of time on mass of ion (Na or SO_4_) accumulated per gram bug mass (GraphPad Prism v9.4.0, GraphPad Software, La Jolla, CA, USA). Mass-specific calculations were based on wet mass. We applied appropriate corrections for spill-over and quench, and only measurements with lumex values <5% and error rates <10% were used in analyses. Linear regressions were performed to analyze the relationship between treatment and ion flux rates using GraphPad Prism (v9.4.0, GraphPad Software).

#### Osmolality

Five *N. triangulifer* nymphs were collected, blotted dry and weighed, then placed in a 1.5 ml microcentrifuge tube, flash frozen and kept in a −80°C freezer for 1 week. Samples were then homogenized. Briefly 30 µl of deionized water was added to the microcentrifuge tube, then samples were crushed with a plastic micropestle. After being briefly (∼3 min) heated in a 70°C water bath, samples were spun in a centrifuge for 10 min. The supernatant was then transferred to a fresh microcentrifuge tube. Osmolality of each sample was measured in duplicate 10 µl aliquots of supernatant using a VAPRO^®^ vapor pressure osmometer (Wescor^®^, Logan, UT, USA), calibrated with 100 and 290 mmol kg^−1^ standards. Data were given in mmol kg^−1^, then corrected to account for the measured osmolality of dilution water and sample mass. A one-way ANOVA followed by Tukey's multiple comparisons test was used to test whether any differences in osmolality occurred between treatments (GraphPad Prism v9.4.0, GraphPad Software).

#### Gene expression

Gene expression at the whole-organism level was assessed by placing nymphs into sterile microcentrifuge tubes and immediately flash freezing them in liquid nitrogen. Five replicates of two individuals were taken at each sampling point (21, 11.7, 8.5, 5.4 and 1.6 kPa). We used two genes previously shown to be responsive to physiological hypoxia in this species, *egl-9* and *ldh* (Chou et al., 2018; Kim et al., 2017), the oxygen sensing gene *gcy-88e*, as well as 9 other genes associated with osmoregulation (see Table 1). *Tubulin* (*tub*) was used as a housekeeping gene for reference (Table 1). Primers were verified through NCBI BLAST to ensure high similarity (>90%) between other more established species (e.g. *A. aegypti*, *D. melanogaster*) and the newly annotated *N. triangulifer* genome*.* Total RNA was isolated from each replicate using the SV Total RNA Isolation System (Promega, Madison, WI, USA) according to the manufacturer's protocol and quantified on a NanoDrop™ 1000 (Thermo Fisher Scientific, Waltham, MA, USA). Then, first-strand cDNA was synthesized from 1 µg of RNA from each sample by MultiScribe™ MuLV reverse transcriptase using random primers (Applied Biosystems, Carlsbad, CA, USA) in 20 µl reactions using a Bio-Rad iCycler (Bio-Rad, Hercules, CA, USA). qPCR was performed on a QuantStudio™ 3 (Thermo Fisher Scientific) using SYBR^®^ Green Supermix (Bio-Rad) in 10 µl reactions with technical triplicates. Custom parameters were used: 2 min at 50°C, 10 min at 95°C, followed by 40 cycles of 95°C for 15 s and 60°C for 1 min. Finally, a melt curve was calculated for each well to ensure sufficient quality of the samples. The delta CT method (Pfaffl, 2001) was used to analyze the relative expression of each amplicon. Expression levels of *tub* were approximately equal across all treatments and were used to normalize results. Primer efficiencies were included in the calculations to normalize expression. Differences among treatments in gene expression were assessed using a one-way ANOVA and Tukey's multiple comparisons test. All treatments were compared with the control (21 kPa) group to quantify differences of gene expression, unless otherwise stated.

**Table 1.**
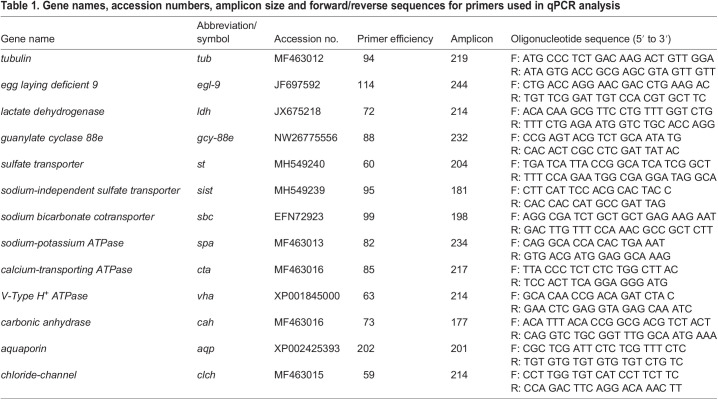
Gene names, accession numbers, amplicon size and forward/reverse sequences for primers used in qPCR analysis

## RESULTS

### Ion flux

There were no observed mortalities when nymphs were exposed to gradients in oxygen. *Ṁ*_O_2__ decreased with lower *P*_O_2__, with the *P*_crit_ occurring somewhere between 1.6 and 8.5 kPa for all 31 individuals at 22°C in Cochran et al. (2021) (Fig. 2A). Na uptake rates (Fig. 2B) and SO_4_ uptake rates (Fig. 2C) were reduced in treatments below 11.7 kPa. At 11.7 kPa, Na uptake was 19% decreased relative to that at 21 kPa (*P*=0.0571; Fig. 2B) and SO_4_ uptake was decreased 51% (*P*=0.0175; Fig. 2C) relative to that at 21 kPa (*P*=0.0128; Fig. 2C). Individuals continuously exposed to all of the preceding conditions of this *P*_O_2__ ramp had marked reductions in their ion uptake rates relative to individuals that were immediately transferred to each condition at 8.5 kPa and below (gray circles versus black squares in Fig. 2B,C).

For individuals only in the experimental water for the 3 h exposure (immediately exposed) at 8.5 kPa, Na uptake was 39% decreased relative to that at 21 kPa (*P*=0.0080; Fig. 2B) and SO_4_ uptake was 68% decreased relative to that at 21 kPa (*P*=0.0077; Fig. 2C). At 8.5 kPa, Na and SO_4_ uptake in continuously exposed nymphs was 33% (*P*=0.0024; Fig. 2C) and 40% (*P*=0.0020; Fig. 2C) lower than uptake in immediately exposed nymphs, respectively. At 5.4 kPa, Na uptake was 60% decreased relative to that at 21 kPa (*P*<0.0001; Fig. 2B) and SO_4_ uptake was 82% decreased relative to that at 21 kPa (*P*<0.0001; Fig. 2C) for immediately exposed nymphs. Na and SO_4_ uptake in continuously exposed nymphs was 62% (*P*<0.0001; Fig. 2B) and 66% (*P*<0.0001; Fig. 2C) lower than uptake in immediately exposed nymphs, respectively. At 1.6 kPa, Na uptake was 72% decreased relative to that at 21 kPa (*P*<0.0001; Fig. 2B) and SO_4_ uptake was 82% decreased relative to that at 21 kPa (*P*<0.0001; Fig. 2C) for immediately exposed nymphs. At 1.6 kPa, Na and SO_4_ uptake in continuously exposed nymphs was 75% (*P*<0.0001; Fig. 2B) and 93% (*P*<0.0001; Fig. 2C) lower than uptake in immediately exposed nymphs, respectively.

### Osmolality

**Fig. 3.**
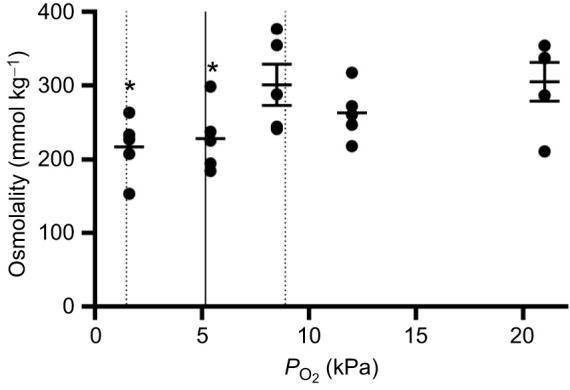
***Neocloeon triangulifer* osmolality across a range of *P*_O_2__ (21–1.6 kPa).** Data are means±s.e.m. (*n*=5). Dashed lines represent maximum and minimum *P*_crit_ estimates and the solid line represents mean *P*_crit_ from Cochran et al. (2021). Differences among treatments were assessed using a one-way ANOVA and Tukey's multiple comparisons test. All treatments were compared with the control (21 kPa) group. Asterisks represent significant differences compared with 21 kPa (*P≤0.05).

An unpaired *t*-test showed a significant decrease in osmolality at and below *P*_crit_ (5.4 and 1.6 kPa; *P*=0.0361 and *P*=0.0244, respectively; Fig. 3). At 5.4 kPa, the osmolality was 25% lower than at 21 kPa and at 1.6 kPa the osmolality was 29% lower relative to that at 21 kPa.

### Gene expression

**Fig. 4.**
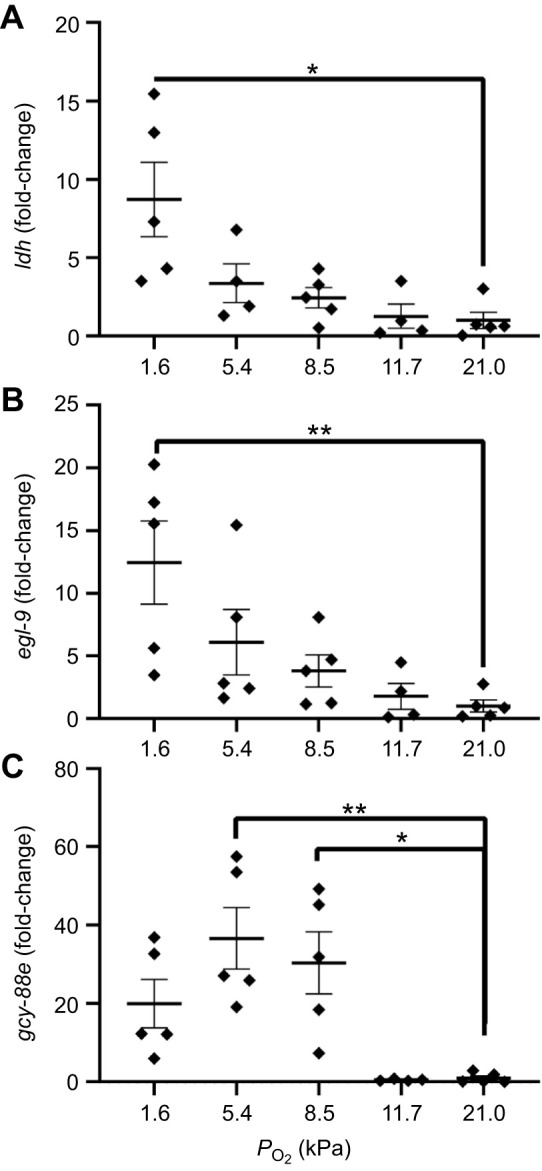
**Expression of hypoxia-associated genes in *N. triangulifer* across a range of *P*_O_2__.** Relative expression of (A) *ldh*, (B) *egl-9* and (C) *gcy-88e* after a 3 h exposure to different oxygen levels at 22°C. All data were normalized to expression of the housekeeping gene *tubulin*, and are expressed as fold-change relative to control samples (means±s.e.m., *n*=5 replicates of 2 individuals per replicate). Differences among treatments in gene expression were assessed using a one-way ANOVA and Tukey's multiple comparisons test. All treatments were compared with the control (21 kPa) group. Asterisks represent significant differences compared with 21 kPa (*P≤0.05; **P≤0.01).

The mRNA levels of nymphs at oxygen saturations above *P*_crit_ were not different from those of control (21 kPa) animals for *ldh* and *egl-9*. However, in the most hypoxic condition, nymphs had elevated expression for *ldh* (1.6 kPa, *P*=0.03; Fig. 4A) and *egl-9* (1.6 kPa, *P*=0.007; Fig. 4B).

At maximum and mean *P*_crit_, nymphs had elevated expression of g*cy-88e* relative to control (21 kPa) animals (8.5 kPa, *P*=0.04; 5.4 kPa, *P*=0.007; Fig. 4C). Below *P*_crit_, expression of *gcy-88e* was not statistically different from that of control animals (1.6 kPa, *P*=0.4; Fig. 4C).

**Fig. 5.**
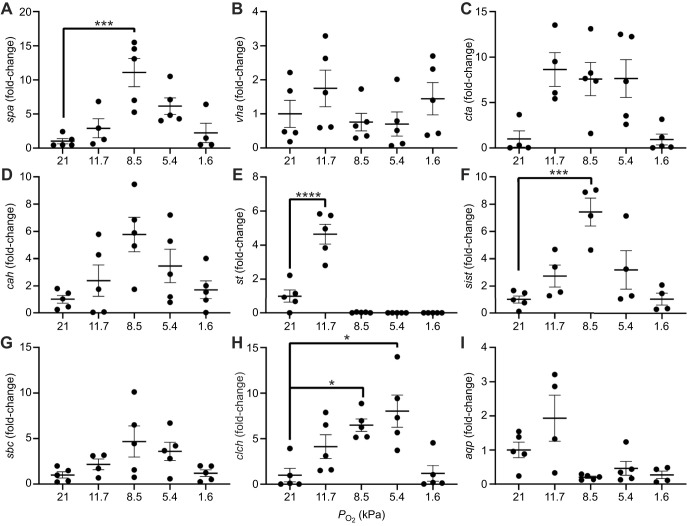
**Expression of ion transport genes in *N. triangulifer* across a range of *P*_O_2__.** Relative expression of (A) *sodium-potassium ATPase* (*spa*), (B) *V-Type H^+^ ATPase* (*vha*), (C) *calcium-transporting ATPase* (*cta*), (D) *carbonic anhydrase* (*cah*), (E) *sulfate transporter* (*st*), (F) *sodium-independent sulfate transporter* (*sist*), (G) *sodium bicarbonate cotransporter* (*sbc*), (H) *chloride-channel* (*clch*) and (I) *aquaporin* (*aqp*) after a 3 h exposure to different oxygen levels at 22°C. All data were normalized to expression of the housekeeping gene *tubulin*, and are expressed as fold-change relative to control samples (means±s.e.m., *n*=4–5 replicates of 2 individuals). Differences among treatments in gene expression were assessed using a one-way ANOVA and Tukey's multiple comparisons test. All treatments were compared with the control (21 kPa) group. Asterisks represent significant differences compared with 21 kPa (*P≤0.05; ***P≤0.001; ****P≤0.0001).

Nymphs at oxygen saturations below control (21 kPa) varied in their mRNA levels of several other genes of interest. While some interesting trends in gene expression were observed, only *sodium-potassium ATPase* (*spa*) (*P*=0.0002; Fig. 5A), *sulfate transporter* (*st*) (*P*<0.0001; Fig. 5E), *sodium-independent sulfate transporter* (*sist*) (*P*=0.0002; Fig. 5F) and *chloride-channel* (*clch*) (8.5 kPa, *P*=0.0103; 5.4 kPa, *P*=0.0012; Fig. 5H) showed statistically significant upregulation with decreased *P*_O_2__.

## DISCUSSION

ATP turnover is reduced as animals are exposed to significant time in hypoxia and anoxia, leading to a cascade of effects including eventual cell death (Boutilier and St-Pierre, 2000). Therefore, we hypothesized that energetically expensive ion uptake would be suppressed when ATP production was suppressed, at partial pressures below *P*_crit_. This is supported by previous studies looking into the osmorespiratory compromise (the trade-off between the demands of high gill permeability for respiration and low permeability for osmoregulation) in freshwater fish. Hypoxia-tolerant freshwater species such as the oscar (Wood et al., 2009, 2007), common killifish (Giacomin et al., 2020; Wood et al., 2019) and tambaqui (Robertson et al., 2015) reduce sodium and water fluxes within an hour of exposure to severe hypoxia, but recover within an hour of return to normoxia. Less hypoxia-tolerant species such as trout (Iftikar et al., 2010; Onukwufor and Wood, 2018) and zebrafish (Onukwufor and Wood, 2020) show elevated sodium and water fluxes at the start of acute hypoxia, but regulate both back towards control levels as hypoxia exposure continues and after return to normoxic conditions (Wood and Eom, 2021). However, in our study we observed significant impacts on uptake of both Na and SO_4_ at partial pressures well above *N. triangulifer*’s *P*_crit_ at 22°C (Fig. 2). We posit that *N. triangulifer* could sense the modest decrease in *P*_O_2__ and therefore conserved energy by limiting ion turnover.

While we observed changes in ion uptake with immediate decreases in *P*_O_2__, we only observed statistically significant changes in osmolality at and below *P*_crit_ (Fig. 3). In previous studies in *N. triangulifer*, total body salts (Cochran and Buchwalter, 2022), sulfur (Buchwalter et al., 2019) and sodium (Scheibener et al., 2016) were strongly regulated despite changes in ion uptake rates. Our findings suggest that in individuals at and below *P*_crit_, reduced ion uptake was at least transiently not offset by increased ion retention or reductions in diffusive ion loss (see Cochran et al., 2024). This could result in ionic dysregulation as a significant consequence of hypoxia in this species. Notably, with the osmolality measurements being from whole-body samples, there are limitations in the interpretation of this data. We suggest that more work is needed to understand whether ionic dysregulation is occurring during hypoxia in this species.

At the level of gene expression, we targeted several genes including some ion transporters and some hypoxia-responsive genes. Many important ion transporters and aquaporins have been identified to date in the mayfly *N. triangulifer*, and our group has previously found changes in *N. triangulifer* mRNA levels and specific proteins associated with salinity exposure (Orr et al., 2023, 2021). Despite seeing interesting trends in the regulation of these genes across oxygen gradients (Table S2), statistically significant upregulation was only seen in *spa*, *st*, *sist* and *clch.*

Na^+^/K^+^-ATPase maintains the ionic gradient across cell membranes and powers cellular functions. Decreases in mRNA expression with increased salinity have been observed in *N. triangulifer* (Orr et al., 2023). Here, *spa* showed gradual upregulation, with statistically significant upregulation occurring at 8.5 kPa, followed by downregulation at/below *P*_crit_. Similar changes were observed in *V-Type H^+^ ATPase*(*vha*), though this change was not statistically significant. V-type H^+^-ATPase functions as a proton pump to aid physiological function of cells (Finbow and Harrison, 1997) and has been linked to ion and water transport in aquatic insects (Nowghani et al., 2017; Weihrauch et al., 2001), with decreased expression in *N. triangulifer* with increased salinity (Orr et al., 2023). Jonusaite and colleagues (2013) found that ion reabsorption was significantly reduced when ion-motive pumps (V-type H^+^-ATPase and Na^+^/K^+-^ATPase) were pharmacologically inhibited. These changes in the regulation of *vha* and *spa* suggest that ion reabsorption may be inhibited at lower *P*_O_2__ and should be further studied.

The trend of immediate stimulation in upregulation with decreases in *P*_O_2__ followed by dramatic downregulation with continued exposure was also observed in *st*, with expression at 11.7 kPa being significantly increased (*P*<0.0001, Fig. 5E). Sulfate transporters are not well understood to date, with some groups proposing that their function is to support reabsorption of essential ions (Markovich and Aronson, 2007) and other groups suggesting that they may be involved in efflux of excess major ions (Orr et al., 2023). We noted a gradual upregulation followed by downregulation at/below *P*_crit_ in *chloride-channel* (*clch*), *sodium bicarbonate cotransporter* (*sbc*), *sist* and *carbonic anhydrase* (*cah*)*.* However, only *clch* and *sist* showed statistically significant elevation.

It would be reasonable to expect that the observed decreases in ion uptake at lower oxygen partial pressures would be accompanied by decreased transporter expression. However, our findings seem to contradict this, with most transporters appearing to increase with lower *P*_O_2__, as ion uptake decreases. We are unsure what led to this interesting finding. One possibility is that internally, ion-deficient tissues are upregulating their ion transport potential. Alternatively, these expression differences could represent the upregulation of transporters on the body surface if the nymphs were preparing themselves to increase transport if they were returned to normoxic conditions. Neither possibility can be substantiated however, and efforts are underway to examine gill-specific mRNA responses to hypoxia in isolated gills. Further, we posit that the observation of upregulation of expression of genes with initial decreases in oxygen and subsequent downregulation of expression with very low oxygen may be associated with upregulation of transcript abundance followed by translation into protein and subsequent depletion of mRNA as oxygen levels decline.

Interestingly, sustained O_2_-sensing signaling has been observed to rely on Ca^2+^ channel relays in *C. elegans* (Busch et al., 2012)*.* Further, carbonic anhydrases are central in mammalian CO_2_ detection and have been associated with CO_2_ detection in fish gills (Hu et al., 2010; Scott, 2011; Tashian, 1989). Studies in *N. triangulifer* have observed differential regulation of these genes and gill proteome in response to salinity challenge (Orr et al., 2023, 2021), but to our knowledge no similar studies exist looking at upregulation of these genes in response to oxygen availability.

Two hypoxia-associated genes have previously been studied in relation to *P*_crit_ in *N. triangulifer*: *ldh* and *egl-9* (Cochran et al., 2021). Our results are commensurate with those of this previous study, with upregulation of both genes occurring below *P*_crit_. Upregulation of *ldh* specifically suggests a switch from aerobic to anaerobic metabolism, which supports the notion that ATP production would be depleted after *P*_crit_, and energetic constraints would be imposed. *egl-9* is an oxygen-sensing prolyl hydroxylase that turns off HIF signaling (Bishop et al., 2004; Epstein et al., 2001; Kim et al., 2017; Semenza, 2001; Shao et al., 2009; Shen and Powell-Coffman, 2003). Studies of the nematode *C. elegans*, the fly *D. melanogaster* and the crustacean *P. clarkii* have shown that HIF is an O_2_-sensing molecule that mediates the response to respiratory gases (De Lima et al., 2021; Ma and Ringstad, 2012). The upregulation of *ldh* and *egl-9* in our results suggest that this mechanism of O_2_ sensing may be occurring below *P*_crit_, but it does not explain the reduction in Na and SO_4_ uptake rates at *P*_O_2__ above *P*_crit_.

*gcy-88e* has also been identified as an oxygen-sensing gene in *C. elegans*, *D. melanogaster* and *P. clarkii* (De Lima et al., 2021; Ma and Ringstad, 2012). In *D. melanogaster*, the GC subunits *gcy-89da*, *gcy-89db* and *gcy-88e* constitute a cyclase that directly binds to O_2_. After binding to O_2_, GCs can convert GTP to cyclic GMP (Ma and Ringstad, 2012). The recent annotation of the *N. triangulifer* genome (NCBI GCF_031216515.1) (O'Leary et al., 2016) allowed us to develop a gene-specific primer for g*cy-88e*. Our results show upregulation of g*cy-88e* at partial pressures approaching and below *P*_crit_ (though data below *P*_crit_ were not statistically significant). This is commensurate with the aforementioned observations and suggests that *N. triangulifer* is able to sense depleted oxygen prior to *P*_crit_. Importantly, we only saw changes in g*cy-88e* expression at 8.5 kPa and below, but saw changes in ion transport and regulation of other genes (e.g. *clch*, *sbc*) prior to these *P*_O_2__. We posit that there may be other oxygen sensing occurring to stimulate those changes prior to upregulation of g*cy-88e*, though we are unsure what those may be. Alternatively, *gcy-88e* may be activated independent of changes in gene expression, with gene expression increasing with delay but protein detecting changes in oxygen earlier on. While we are still uncertain where this sensing is occurring, insects have many chemoreceptors, thermoreceptors and mechanoreceptors which can appear as hairs, campaniform sensilla or chordotonal organs (Keil, 2012; Rebora et al., 2019). Many chemoreceptors have specifically been described in the antennae and gills (Rebora et al., 2019; Wichard et al., 1973).

A recent study of chronic oxygen limitation throughout development in *N. triangulifer* found that as DO decreases, survival, adult mass and instantaneous growth rate decrease while gill size increases (Funk et al., 2021). These changes suggest that the energy budgets for development, reproduction and growth may be reallocated to oxyregulation in limited oxygen environments. More work is needed to understand the chronic impacts of hypoxia on osmoregulation and possible subsequent physiological changes. Further work is also needed to identify the specific locations and mechanisms of oxygen (and salinity) sensing in aquatic insects. It would also be beneficial for future studies to query what other genes may be impacted by depleted oxygen. Questions also remain as to what *P*_O_2__ are associated with the onset of oxygen sensing, and whether *N. triangulifer* nymphs energetically prioritize osmoregulation again when returned to normoxic conditions. This work signifies a first step in elucidating how oxygen is sensed and responded to in *N. triangulifer* nymphs, even in oxygen conditions not associated with anaerobic metabolism.

## Supplementary Material

10.1242/jexbio.247916_sup1 Supplementary information
