# Supplementary material for: The mayfly Neocloeon triangulifer senses decreasing oxygen availability (PO2) and responds by reducing ion uptake and altering gene expression
Source: J Exp Biol. 2024 Nov 28;227(23):jeb247916. doi: 10.1242/jeb.247916 (PMC11634025; doi:10.1242/jeb.247916)
Supplement: Supplementary information [file jexbio-227-247916-s1.pdf]

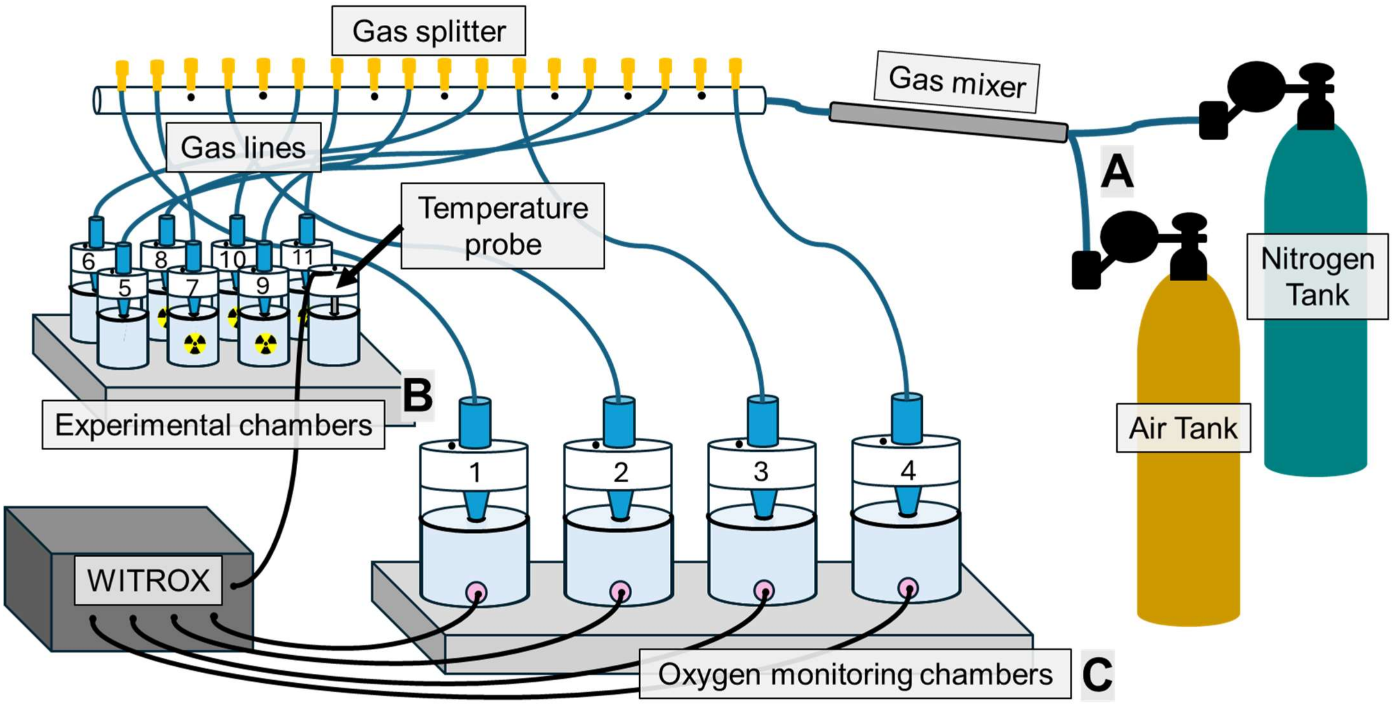

Fig. S1. Diagram of experimental set up. **A)** Air tank and splitter set up, **B)** chamber set up, **C)** monitoring chambers. Chamber numbers directly reflect chamber numbers from supplemental table 1.

Journal of Experimental Biology • Supplementary information

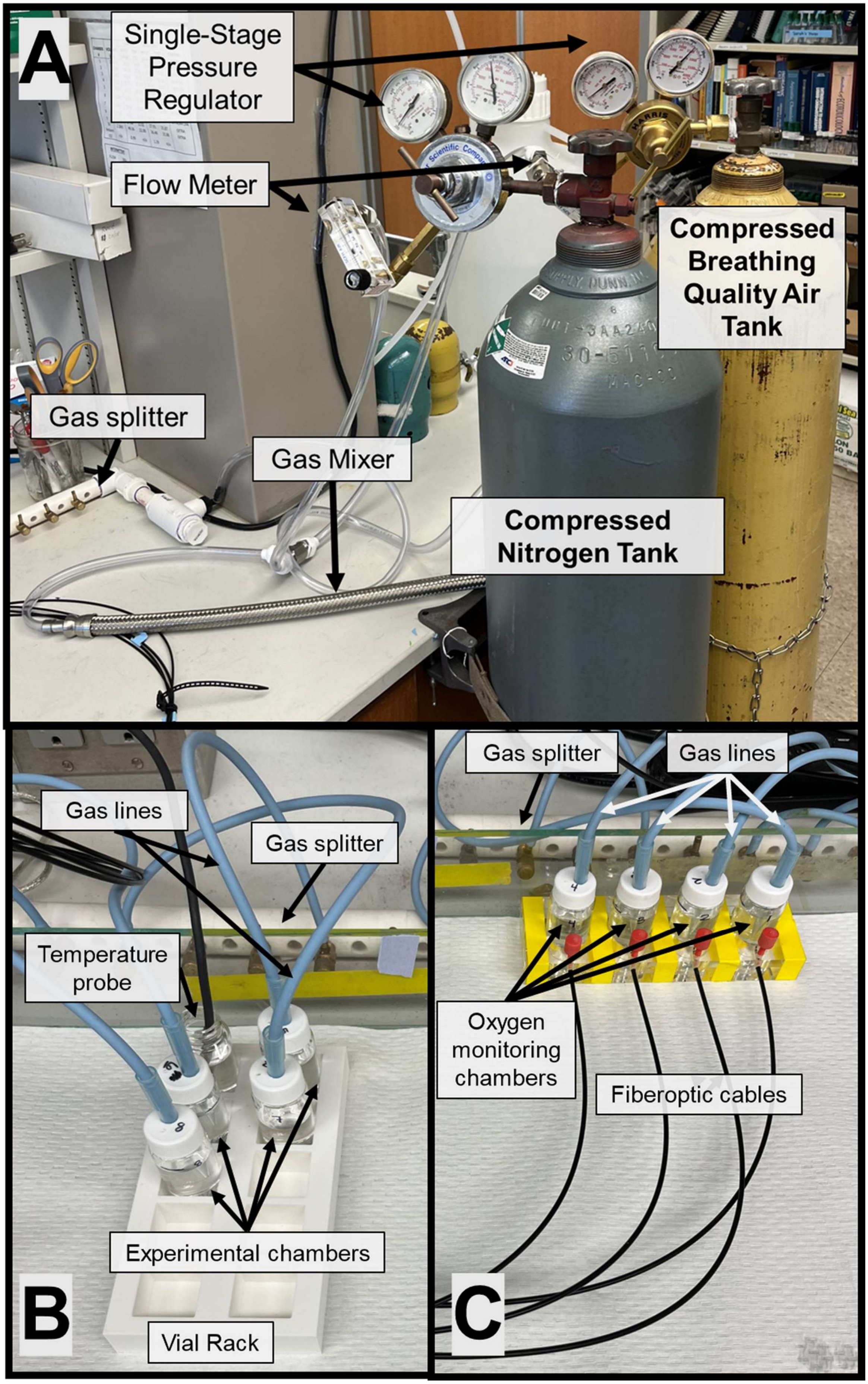
A
Single-Stage Pressure Regulator
Flow Meter
Compressed Breathing Quality Air Tank
Gas splitter
Gas Mixer
Compressed Nitrogen Tank
B
Gas lines
Gas splitter
Temperature probe
Experimental chambers
Vial Rack
C
Gas splitter
Gas lines
Oxygen monitoring chambers
Fiberoptic cables

Fig. S2. Diagram of experimental set up. **A)** Air tank and splitter set up, **B)** an example of chamber set up, **C)** monitoring chambers. The number of chambers here do not directly reflect the total number of chambers used in the experiment but instead serve to reflect how the experiment was reflected. See supplemental table 1 and supplemental figure 1 for exact chamber configuration.

Table S1. Experimental information including whether the oxygen in the chamber was monitored, if radioactive isotope was used in that chamber, immediate or continuous exposure and the change in and total individuals over time. There was also a separate chamber that was unmonitored, non radioactive and was only used to monitor temperature throughout the experiment (see supplemental figure 1 & supplemental figure 2B).

Chamber number	Monitored	Radioactive	Immediate or continuous	A: 21kPa (0h)		B: 21kPa (3h)		C: 11.7kPa (3h)		D: 8.5kPa (0h)		E: 8.5kPa (3h)		F: 5.4kPa (0h)		G: 5.4kPa (3h)		H: 1.6kPa (0h)		I: 1.6kPa (3h)	
				Change	**Total**	Change	**Total**	Change	**Total**	Change	**Total**	Change	**Total**	Change	**Total**	Change	**Total**	Change	**Total**	Change	**Total**
1	Y	N	-	0	**0**	0	**0**	0	**0**	0	**0**	0	**0**	0	**0**	0	**0**	0	**0**	0	**0**
2	Y	N	-	0	**0**	0	**0**	0	**0**	0	**0**	0	**0**	0	**0**	0	**0**	0	**0**	0	**0**
3	Y	N	Continuous	+19	**19**	-4	**15**	-3	**12**	0	**12**	-4	**8**	0	**8**	-4	**4**	0	**4**	-4	**0**
4	Y	N	Continuous	+19	**19**	-4	**15**	-4	**11**	0	**11**	-3	**8**	0	**8**	-4	**4**	0	**4**	-4	**0**
5	N	N	Continuous	+19	**19**	-4	**15**	-4	**11**	0	**11**	-4	**7**	0	**7**	-3	**4**	0	**4**	-4	**0**
6	N	N	Continuous	+18	**18**	-3	**15**	-4	**11**	0	**11**	-4	**7**	0	**7**	-4	**3**	0	**3**	-3	**0**
7	N	Y	Continuous	+10	**10**	-2	**8**	-2	**6**	0	**6**	-2	**6**	0	**6**	-2	**4**	0	**4**	-2	**0**
8	N	Y	Continuous	+10	**10**	-2	**8**	-2	**6**	0	**6**	-2	**6**	0	**6**	-2	**4**	0	**4**	-2	**0**
9	N	Y	Continuous	+10	**10**	-2	**8**	-2	**6**	0	**6**	-2	**6**	0	**6**	-2	**4**	0	**4**	-2	**0**
10	N	Y	Continuous	+10	**10**	-2	**8**	-2	**6**	0	**6**	-2	**6**	0	**6**	-2	**4**	0	**4**	-2	**0**
11	N	Y	Immediate	0	**0**	0	**0**	0	**0**	+8	**8**	-8	**0**	+8	**8**	-8	**0**	+8	**8**	-8	**0**

Journal of Experimental Biology • Supplementary information

Table S2. mRNA transcript levels in *N. triangulifer* after 3-hour exposure to different oxygen levels at 22ºC corresponding with figure 5. All data are normalized to the housekeeping gene, tubulin, and expressed as fold-change relative to control samples. (n=4-5 replicates of 2 individuals).

	**1.6**			**5.4**			**8.5**			**11.7**			**21**		
	**Mean**	**SEM**	**N**	**Mean**	**SEM**	**N**	**Mean**	**SEM**	**N**	**Mean**	**SEM**	**N**	**Mean**	**SEM**	**N**
Ca^{2+}ATPase	0.93	0.60	5	7.64	2.08	5	7.59	1.83	5	8.63	1.86	4	1.00	0.89	4
Carbonic Anhydrase	1.71	0.67	5	3.47	1.22	5	5.748	1.26	5	1.92	1.15	5	1.00	0.30	5
Na^{-}/K^{+}ATPase	2.22	1.41	4	6.15	1.22	5	11.08	2.07	5	2.91	1.39	4	1.00	0.39	5
Na-Ind SO_4^- Transporter	1.04	0.44	4	3.18	1.41	4	7.43	1.02	4	2.73	0.80	4	1.00	0.28	5
$NaHCO_3$Cotransporter	1.19	0.36	5	3.59	0.99	5	4.67	1.71	5	2.17	0.60	4	1.00	0.34	5
Cl-channel	1.18	0.86	5	8.02	1.76	5	6.49	0.69	5	4.12	1.31	5	1.00	0.75	5
V-Type ATPase	1.44	0.47	5	0.70	0.35	5	0.76	0.26	5	1.75	0.53	5	1.00	0.40	5
SO_4^-Transporter	0.00	0.00	5	0.00	0.00	5	0.02	0.01	5	4.64	0.58	5	1.00	0.36	5
Aquaporin	0.27	0.11	4	0.46	0.20	5	0.20	0.03	5	1.93	0.67	4	1.00	0.23	5
